# Achieving the UNAIDS 95-95-95 treatment target by 2025 in Ghana: a myth or a reality?

**DOI:** 10.1080/16549716.2023.2271708

**Published:** 2023-11-03

**Authors:** Dorothy Serwaa Boakye, Samuel Adjorlolo

**Affiliations:** a Department of Health Administration and Education, University of Education, Winneba, Ghana; b School of Nursing and Midwifery, College of Health Sciences, University of Ghana, Legon, Ghana; c Research and Grants Institute of Ghana, Legon, Ghana

**Keywords:** HIV/AIDS response, HIV testing, treatment, viral suppression, achievements, challenges, Ghana

## Abstract

The United Nations Joint Programme on HIV/AIDS (UNAIDS) has set ambitious treatment targets known as Project 95-95-95, aiming to achieve 95% of people living with HIV knowing their status, 95% of diagnosed individuals on antiretroviral therapy (ART), and 95% of those on ART achieving viral suppression by 2025. Through a comprehensive analysis of Ghana’s HIV/AIDS response, we evaluate the feasibility of Ghana’s efforts in realising these targets. The discussion explores Ghana’s achievements in HIV testing and diagnosis, ART coverage, and viral suppression rates, as well as challenges related to stigma, limited access to healthcare services, funding constraints, and data quality. Strategies such as strengthening prevention efforts, expanding access to ART, addressing stigma, and enhancing health systems are discussed as the way forward to advance Ghana’s progress towards the UNAIDS 95-95-95 treatment targets. While Ghana has made significant strides in its HIV/AIDS response, achieving the 95-95-95 targets is a challenging yet realistic goal.

## Introduction

The global fight against HIV/AIDS has been a persistent challenge, but the United Nations Joint Programme on HIV/AIDs (UNAIDS) launched Project 95-95-95 with the ambitious goal of ending the epidemic by 2030 [1,2]. This initiative aims to achieve three critical targets: 95% of people living with HIV knowing their status, 95% of diagnosed individuals receiving antiretroviral therapy (ART), and 95% of those on treatment achieving viral suppression [1,3,4]. Among the countries striving to achieve these targets is Ghana, a West African nation that has made significant progress in addressing HIV/AIDS but still faces substantial challenges.

In Ghana, healthcare predominantly operates within a five-tiered system under the public sector. This system comprises health posts, health centers, and clinics, followed by district hospitals, regional hospitals, and tertiary hospitals. Tertiary, regional, and district hospitals (mainly of which are missions) focus on curative services. Health centers and clinics, alongside outreach programmes, deliver both preventive and curative services to communities within their designated areas [5].

In June 2002, ART for the general population and prevention of mother-to-child transmission (PMTC) was initially launched in Ghana at two pilot sites in the Eastern region [6]. Subsequently, the Ghanaian government, in partnership with development partners and civil society organisations, implemented various initiatives to prevent new infections, expand testing and treatment coverage, and enhance the overall well-being of individuals living with HIV/AIDS [7,8]. Supported by funding from the Global Fund to fight AIDS, tuberculosis, and malaria, Ghana significantly expanded its treatment and counselling services nationwide in 2005. The number of treatment centres rose from 2 in 2003 to 197 across 145 districts by 2018 [9]. Presently, HIV treatment and prevention services, including education, contraceptives, PMTC, and pre-exposure prophylaxis (PrEP), are offered by teaching, regional and district health facilities, Christian Health Association of Ghana, and selected private healthcare facilities [10]. Ghana’s healthcare system relies heavily on international financial and technical aid. While the National Health Insurance Scheme (NHIS) covers symptomatic HIV/AIDS treatment for opportunistic infections, it expressly excludes ART medication [11]. However, ART is predominantly funded by the National AIDS Programme and is provided free of charge at public facilities when available [12].

To enhance Ghana’s progress in achieving the 90-90-90 treatment target in 2020, the National AIDS/STI Control Programme (NACP) embraced the 2016 WHO guidelines, ensuring all individuals living with HIV/AIDS receive ART regardless of immune status or clinical stage. One of the adopted strategies was Differentiated Service Delivery (DSD), which aimed at improving HIV service provision and expediting the national HIV response [13]. Although the implementation of the DSD-based acceleration plan led to significant advancements in Ghana’s HIV programme, the nation fell short of the United Nations Programme on HIV/AIDS 90-90-90 targets. By the end of 2020, 63% of the individuals knew their HIV status, 95% were on ART, and 73% achieved viral suppression [13]. Ghana's national strategic plan for HIV 2021–2025 aims to achieve the ambitious 95-95-95 target by 2025, focusing on critical areas such as improving access to healthcare [14]. A fresh emphasis on DSD Strategic Initiative (DSD SI) for individuals with HIV strives to tailor health services in person-centred ways while ensuring the healthcare system is not overburdened [15]. These efforts have yielded positive outcomes, including increased awareness, improved access to ART, and a reduction in new infections over the years [6,16]. For instance, the national HIV incidence among adults has decreased by 5% from 2004 to 2020 [17]. Moreover, a report from WHO [15] also showed that linkage to ART improved from 85% to 99% in a polyclinic in the Greater Accra region a year after the DSD SI initiative.

However, several challenges persist, raising questions about the feasibility of achieving the UNAIDS treatment target by 2025. Stigma and discrimination remain pervasive, hindering individuals from seeking testing and treatment services and affecting their overall quality of life [18,19]. Access to healthcare, especially in rural and remote areas, remains limited, impacting timely diagnosis and comprehensive care [20,21]. Additionally, the need for sustained funding and resources to support the HIV/AIDS response remains a critical concern [7,22].

Furthermore, the success of achieving the UNAIDS treatment target in Ghana depends on various factors. These include strengthening HIV prevention efforts, addressing social and structural barriers, enhancing the reach and quality of HIV testing services, improving treatment adherence and retention, and ensuring sustainable funding and political commitment. Collaborative efforts among government agencies, healthcare providers, civil society organisations, affected communities, and international partners are crucial to surmounting these challenges and achieving the desired targets [23–25].

Considering the notable success stories and the challenges glaring Ghana in the face, the following question arises: is achieving this ambitious target by UNAIDS in Ghana by 2025 a myth or a reality? To explore this question, it is crucial to evaluate the progress made by Ghana in reaching the 95-95-95 targets and identify the challenges that hinder their realisation. Understanding the current situation will help policymakers, healthcare professionals, and stakeholders develop effective strategies to address the gaps and work towards achieving UNAIDS treatment targets.

This paper will critically analyse the existing evidence, interventions, and strategies employed by Ghana in its HIV/AIDS response, weighing the achievements against the remaining gaps and challenges. We also aimed to provide insights and recommendations to strengthen the country’s efforts and accelerate progress towards ending the epidemic.

## Prevalence of HIV/AIDS in Ghana

The national HIV prevalence rate is recorded at 1.67% [26], with varying rates across regions. The Eastern Region has the highest prevalence at 2.3%, followed closely by the Greater Accra Region and the Western Region at 2.1% and 2.2%, respectively. In contrast, the Northern Region reports the lowest rate, with only 0.2% of the population affected by HIV/AIDS. There is a notable gender disparity, with 55% of people living with HIV being women. Young people aged 15 to 24 constitute 25% of the HIV-positive population in the country [7,27]. Among key populations, the prevalence rates are significantly higher, with 18.1% among men who have sex with men (MSM) and 6.9% among female sex workers (FSW) [28,29]. Within the first half of 2022, the National STIs and HIV/AIDS Control Programme have recorded 23,495 new infections [30].

## Ghana’s progress in HIV testing and diagnosis

Ghana has taken significant steps to increase HIV testing rates, a critical component in achieving the UNAIDS treatment target. According to Giguère et al. [31], timely and accurate testing is essential to identify individuals living with HIV, link them to care and treatment, and prevent further transmission. The country has implemented various innovative approaches, including community-based testing, mobile testing units, and self-testing kits, to reach individuals who may face barriers in accessing traditional healthcare settings. Community-based testing and mobile testing were initiated in the country in 2016 following the adoption of WHO’s DSD. These efforts have resulted in expanded testing coverage, improved detection of HIV cases and linkage to care, contributing to the overall goal of the 95-95-95 targets [22,23,32,33]. In June 2022, Ghana officially introduced and implemented an HIV self-testing programme, empowering its citizens to assess their HIV status confidentially and conveniently within the confines of their homes. According to the Ghana AIDS Commission [34], the national HIV testing coverage significantly rose to 72% by 2022, a notable increase from 69% in 2019 and 63% in 2020, indicating considerable improvements. However, across the country, testing is suboptimal among people at high risk of HIV infection. In a 2019 nationwide survey of FSWs, only 56.5% had tested for HIV in the previous 12 months [35]. Testing coverage among MSM was only 26.6% in the 12 months preceding the 2017 Ghana Men Study II [29]. There has been a general downward trend in testing coverage among pregnant women over the last five years: 80% in 2015, 76% in 2016, 71% in 2017, 73% in 2018, and 72% in 2019 [36].

To strengthen testing services, Ghana has emphasised the integration of HIV testing with other essential health services. This integration approach includes incorporating HIV testing into antenatal care, tuberculosis screening, and sexual and reproductive health services. Ghana aims to increase testing opportunities and reach populations that may not actively seek HIV testing by integrating testing into existing healthcare platforms [37,38].

Furthermore, Ghana has made progress in implementing provider-initiated testing and counselling (PITC) in healthcare settings. PITC involves healthcare providers offering HIV testing to all individuals attending healthcare facilities, regardless of their presenting symptoms or reasons for seeking care [24]. This approach helps normalise HIV testing, reduces stigma, and ensures that more individuals are aware of their HIV status. The adoption of PITC has resulted in increased HIV case detection and earlier linkage to care and treatment [23].

Despite these achievements, Ghana faces several challenges in HIV testing and diagnosis. Stigma and discrimination remain significant barriers, deterring individuals from seeking HIV testing services [22,39]. Fear of social consequences, including disclosure and potential discrimination, often prevents people from accessing testing facilities or disclosing their HIV status to healthcare providers. Addressing stigma and discrimination through community engagement, education, and advocacy is crucial to improving testing rates and the overall HIV response [25].

Another challenge is the limited availability of testing services, particularly in rural and remote areas. Access to healthcare facilities and testing centres remains a concern, leading to disparities in testing coverage. To address this, Ghana has implemented mobile testing units and community-based testing programmes to reach underserved populations. These strategies aim to bring testing services closer to communities, reduce transportation barriers, and improve access to testing in remote areas [7].

To enhance HIV testing and diagnosis, Ghana has also invested in training healthcare workers to provide quality counselling and testing services [32]. Ensuring competent and compassionate healthcare providers is crucial to creating a supportive environment for individuals seeking testing. By strengthening healthcare provider capacity, Ghana aims to improve the overall testing experience and increase the testing rates.

## Antiretroviral therapy coverage: Ghana’s progress so far

Ghana has made significant progress in expanding ART coverage, which plays a crucial role in improving the health outcomes and quality of life of people living with HIV/AIDS. ART is a cornerstone of HIV treatment, suppressing the virus, and reducing the risk of transmission [16]. This section delves into Ghana’s progress in ART coverage, highlighting achievements, challenges, and strategies employed to expand access to treatment.

Ghana has demonstrated commendable efforts in scaling up ART coverage over the years. The country has implemented policies and programmes to improve access to treatment services, resulting in increased ART coverage. Through collaboration with various stakeholders, including government agencies, healthcare providers, civil society organisations, and international partners, Ghana has successfully expanded the reach of ART services [23]. In 2021, 73% of the individuals who were aware of their HIV status were receiving life-saving ART, a decline from the 78% recorded in 2020 [40]. However, by the close of 2022, ART coverage had increased to 87% [34]. Notably, coverage among specific key groups such as MSM, FSWs, and prisoners remained high at 95.1%, 99%, and 100%, respectively, in 2022 [26]. ART coverage displayed progress across different age categories: in adults aged 15 and above and in children (0–14), the coverage rates increased from 27.87% and 12.05% in 2015 to 33.88% and 16.94% in 2016, steadily rising to 41.83% and 23.05% in 2017. Pregnant women witnessed significant improvements in ART coverage due to the PMTC) services, with rates of 42.68%, 53.33%, and 65.97% in 2015, 2016, and 2017, respectively [41].

One key strategy implemented in Ghana is the decentralisation of ART services. The government has worked to establish ART sites across the country, including in rural and remote areas. By bringing treatment closer to communities, Ghana has reduced geographical barriers and improved access to ART for individuals who previously faced challenges in reaching healthcare facilities. Decentralisation has significantly contributed to the expansion of ART coverage and improved health outcomes [23]. While Ghana's HIV programme has made progress in reaching more people, large treatment gaps remain; nationwide, some 40% of the people with HIV have not started lifesaving ART, indicating a need to improve linkage from testing services to treatment services. For instance, of the 18% of MSM living with HIV/AIDS, only 3.7% have been linked to care and are receiving treatment. Furthermore, paediatric ART coverage is minimal (less than 30%) [42]. Nevertheless, for the majority of PLWHA on treatment, 79% of them have their virus suppressed, indicating programme strength in supporting adherence to ART [15,43].

Task-shifting and task-sharing have played a vital role in expanding ART coverage in Ghana. The country has trained and empowered a broader range of healthcare workers, such as nurses and community health workers, to initiate and manage ART. This strategy has helped address human resource limitations and enabled more individuals to receive treatment. By involving different cadres of healthcare providers, Ghana has expanded its capacity to deliver ART services and reached underserved populations [23].

Ghana has also adopted a patient-centred approach to improve ART retention and adherence. The country has implemented community-based adherence support programmes, peer support networks, and innovative interventions such as mobile phone reminders. These strategies aim to address the barriers that individuals face in adhering to treatment, including stigma, transportation challenges, and medication adherence. By providing ongoing support and tailored interventions, Ghana has improved treatment retention rates and achieved better health outcomes for individuals on ART [23,44].

Despite progress, challenges persist in expanding ART coverage in Ghana. Stigma and discrimination remain significant barriers, leading to delays in seeking treatment and hindering adherence to ART [22]. To address this, Ghana has implemented stigma reduction initiatives, such as community education programmes, and campaigns to raise awareness and promote acceptance of people living with HIV/AIDS. Additional initiatives aimed at PLWHA include psychoeducation, counselling, skills development, and a web-based short message reporting system to address instances of stigma within healthcare facilities [7,8,45]. By combating stigma, Ghana aims to improve treatment-seeking behaviour and increase ART coverage [6]. Furthermore, the poor quality of ART services rendered to people living with HIV/AIDS (PLWHA) is partly to blame for Ghana’s unmet targets for ART coverage and performance. Particularly, the main constraints to providing optimal quality ART service are inadequate medications and service providers demanding money from PLWHA for payment of antiretrovirals, lack of viral load (VL) machines and reagents for CD4 test [46].

Sustained funding for ART services is another challenge. Ghana, in collaboration with international partners, has worked towards ensuring a sustainable financing mechanism for the procurement, distribution, and monitoring of antiretroviral medications. Advocacy for increased domestic funding and exploring innovative financing strategies are crucial to maintaining and expanding ART coverage in the long term [25].

## Ghana’s progress with viral suppression rates

Ghana has made notable progress in achieving viral suppression rates among individuals living with HIV/AIDS. Viral suppression is a critical indicator of successful treatment and plays a crucial role in reducing HIV transmission. This chapter will focus on Ghana’s progress in achieving viral suppression rates, highlighting statistics and the strategies employed to improve outcomes.

According to the Ghana AIDS Commission, in the early part of 2021, the viral suppression rate among individuals receiving ART in Ghana is estimated to be approximately 81% [7]. As of December 2021, the percentage with evidence of viral suppression had declined to 79% [47]. Nevertheless, these statistics reflect the proportion of individuals on ART who have achieved and maintained viral suppression, defined as having a very low or undetectable level of HIV in their blood. The 79% is an improvement of the 66% and 68% recorded in 2018 and 2019 respectively [36].

Ghana has implemented various strategies to improve viral suppression rates. These strategies include ensuring widespread access to ART, promoting adherence to treatment regimens, and providing ongoing support to individuals living with HIV/AIDS. The country has focussed on decentralised healthcare delivery, establishing ART sites across the country to bring treatment closer to communities and improve access [38].

Furthermore, Ghana has implemented patient-centred approaches to enhance adherence and retention in treatment. Community-based adherence support programmes, peer support networks, and mobile phone reminders are among the initiatives employed to provide ongoing support and improve treatment adherence. These efforts have contributed to better viral suppression rates by ensuring that individuals consistently take their medication as prescribed [7,23].

The implementation of the ‘test and treat’ approach, where individuals diagnosed with HIV are immediately initiated on ART regardless of CD4 cell count or clinical stage, has also played a significant role in improving viral suppression rates [23]. Moreover, the incorporation of the WHO ‘test and treat’ policy facilitated the introduction of PITC, client-initiated testing, and nurse-initiated ART treatment (as per Ghana’s task shifting policy). This approach ensured that individuals testing positive for HIV were eligible for ART on the same day, leading to increased acceptance and uptake of treatment services [43]. Early initiation of treatment helps suppress the VL and reduces the risk of transmission [48].

Despite progress, challenges exist in achieving optimal viral suppression rates. Stigma, discrimination, and psychosocial factors can impact treatment adherence and retention, affecting viral suppression outcomes. Addressing these challenges requires comprehensive approaches that include awareness campaigns, community education, and support networks to combat stigma and promote acceptance [22,49].

It is crucial to continuously monitor and evaluate viral suppression rates to assess progress and identify areas for improvement. Meeting the increased demand for VL testing is also crucial to achieve the UNAIDS set target for viral suppression. Establishing efficient specimen referral networks and reinforcing laboratory capacities are vital steps in expanding VL testing services. Ghana Health Service put the specimen referral network into action by negotiating an agreement with Ghana Post Company Ltd to provide third-party courier services for sample transport and delivery of results to referring facilities for decision-making. The Ghana Post Company Ltd. was used to spread out the specimen referral system to all ART facilities in Ghana’s 16 regions. Its deployment has increased HIV VL test coverage by approximately 71% (from 54,538 in 2018 to 93,013 in 2019) [36]. Furthermore, regular data collection, analysis, and feedback mechanisms help to inform policy decisions, target interventions, and allocate resources effectively. Ghana’s commitment to data-driven decision-making will contribute to ongoing efforts to improve viral suppression rates [50].

## Challenges and barriers hindering Ghana’s efforts towards the achievement of the UNAIDS treatment target

Ghana has made significant progress in its HIV/AIDS response, but several challenges and barriers hinder its progress towards achieving the 95-95-95 targets set by UNAIDS. Understanding these challenges is crucial for developing effective strategies to overcome them.

1. Stigma and Discrimination: stigma and discrimination remain pervasive issues in Ghana, affecting individuals living with HIV and key affected populations. Fear of disclosure and social consequences led to reluctance to seek HIV testing, care, and treatment services. Stigma also affects treatment adherence and retention, hindering progress towards achieving viral suppression rates [7,22,51].
2. Limited Access to Healthcare Services: accessibility to HIV testing, treatment, and care services remains a challenge, particularly in remote and rural areas. In addition, prioritisation of HIV testing services to achieve the greatest yield of new diagnoses is one of the major challenges faced by testing programmes [52] in Ghana. Limited infrastructure, long distances to healthcare facilities, and inadequate transportation contribute to barriers to accessing essential services. This disparity in access hampers progress towards achieving the 95-95-95 targets, especially in underserved populations [7].
3. Funding Constraints: sustained funding for HIV/AIDS programmes is crucial to achieving the 95-95-95 targets. Ghana, like many countries, faces challenges in securing adequate and sustainable funding for the comprehensive HIV/AIDS response. Reliance on external funding sources and competing priorities within the healthcare sector pose challenges in implementing and scaling up effective interventions [8,50].
4. Retention in Care and Adherence to Treatment: maintaining individuals on treatment and ensuring high levels of adherence to ART regimens are essential for achieving viral suppression. Challenges such as treatment fatigue, side effects, and logistical barriers can impact adherence rates. Additionally, psychosocial factors, including mental health issues and lack of support systems, may contribute to lower retention rates [7].
5. Key Populations (KP) and Vulnerable Groups: key populations, including men who have sex with men, sex workers, people who inject drugs, and transgender individuals, often face heightened vulnerabilities and barriers to accessing HIV services due to societal stigma, discrimination, and legal frameworks. For instance, a systematic review and meta-analysis have shown that among African men who have sex with men, lower testing and knowledge of status were associated with hostile legislation [52]. Moreover, there are limited data specific to ART coverage and viral suppression rates among key populations in Ghana [53]. The Ghana Demographic and Health Survey, a primary source of HIV data in the country, rarely includes information on key populations [54]. Ali et al. [55] pointed out the absence of data regarding the number of HIV-positive individuals within key populations who are linked to care and treatment. Laar & DeBruin [56] highlighted a significant reason for this deficiency in data collection among key populations. According to the authors, institutional stigma and discrimination hinder the regular access of key populations to necessary care and treatment [56]. Addressing the specific needs of these populations and ensuring their inclusion in HIV/AIDS programmes are crucial for achieving the 95-95-95 targets [8].

## Recommendations and strategies for enhanced efforts towards the achievement of UNAIDS 95-95-95 treatment targets

To advance Ghana’s efforts in achieving the UNAIDS treatment target, several strategies and approaches can be pursued. These strategies encompass various aspects of HIV/AIDS response, including testing, treatment, prevention, and the elimination of barriers to access. This part of the discussion explores some of the key strategies and the way forwards for Ghana:

1. Scaling Up HIV Testing Services: strengthening and expanding HIV testing services are crucial to identify individuals living with HIV and link them to care and treatment [23]. Innovative approaches, such as community-based testing, self-testing kits, and integration of HIV testing with other healthcare services, can be employed to increase testing coverage and reach underserved populations. Mobile testing units and targeted testing in key populations can also help improve testing rates [7,8]. Self-testing kits can be made accessible over-the-counter at pharmacies, drug stores, or through online retailers. Within the communities, health workers can utilise home-based testing in the privacy of the individual’s home [33,57]. Mobile apps, telehealth platforms, and hotlines can be utilised to provide guidance and support aiding in linkage to care for individuals using self-test kits [58].
2. Enhancing Treatment Access and Retention: improving access to ART and retention in care are essential for achieving the treatment target. Ghana can continue decentralising ART services to bring treatment closer to communities, particularly in rural and remote areas [7]. Implementing DSD models, such as multi-month scripting and community-based ART distribution, can also enhance treatment access and retention [8,46].
3. Addressing Stigma and Discrimination: efforts to combat stigma and discrimination are critical to improving HIV service utilisation and outcomes. Ghana should invest in comprehensive stigma reduction programmes that include community education, awareness campaigns, and engagement with key stakeholders, including healthcare providers and community leaders [7]. Promoting inclusive policies and legal reforms that protect the rights of key populations is also essential [8,22]. A report from WHO [59] suggested the implementation and enforcement of antidiscrimination and protective laws to eradicate stigma and discrimination, training and sensitisation of health workers, and providing HIV-friendly services for key populations to improve HIV health service coverage in Western and Central Africa.
4. Strengthening Health Systems and Workforce: building robust health systems and ensuring an adequately trained and supported healthcare workforce are essential for achieving the treatment target. Ghana can invest in healthcare infrastructure, laboratory services, and supply chain management to support HIV/AIDS programmes [7]. Expanding the capacity of healthcare workers through training and mentorship programmes can improve service delivery and quality of care [8,50]. Laboratory services can include decentralisation of laboratory services to bring testing and monitoring closer to the communities where they are needed, strengthening the supply chain to ensure the availability of reagents, consumables, and test kits for HIV testing and VL monitoring, and implementing a robust quality assurance programme to maintain the accuracy and reliability of laboratory results [33,60].
5. Strengthening HIV prevention efforts: strengthening HIV prevention efforts in Ghana is essential to reduce new infections and curb the spread of the virus. Ghana has implemented various successful prevention strategies, including promoting condom use, education on prevention, advocating for PrEP for high-risk groups like sero-discordant couples, and ensuring access to post-exposure prophylaxis. Coupled with the introduction of ART, these measures have significantly contributed to Ghana’s reduction in new HIV infections. Notably, adult HIV incidence decreased by 5% from 2004 to 2020 [7,17]. Nonetheless, further improvements are needed to strengthen Ghana’s HIV prevention efforts. The Ghana AIDS Commission and the Ministry of Education should collaborate closely to enhance comprehensive sexuality education in schools and communities. This would boost awareness about HIV transmission and prevention among adolescents and young adults. Encouraging open and non-stigmatising discussions about sexual health and behaviour is crucial [61]. Additionally, the Ghana Health Service should expand harm reduction programmes, incorporating initiatives like needle exchange and opioid substitution therapy, to curb HIV transmission among people who inject drugs [62].
6. Engaging Communities and Key Populations: meaningful engagement of communities, including key populations, is crucial for a successful HIV/AIDS response. Ghana should foster partnerships with civil society organisations and affected communities to ensure their active involvement in programme design, implementation, and monitoring [7,25]. Tailored interventions for key populations, including targeted outreach and peer support networks, can enhance their access to and uptake of HIV services [8]. The rollout of the LGBTIQ inclusion index which aims to measure the inclusion of LGBTIQ people across five areas: health, education, personal safety and violence, civil and political participation, and economic empowerment [25] will go a long way to end the health inequities that exist among this population in Ghana.
7. Ensuring Sustainable Funding: adequate and sustained funding is vital to support Ghana’s HIV/AIDS programmes and achieve the treatment target. Ghana should advocate for increased domestic funding by allocating a significant portion of the national budget to HIV/AIDS programmes [7]. International donors provide around 63% of HIV funds in Ghana. Countries such as Namibia, which met the UNAIDS 90-90-90 targets in 2020, receive only 29% of their overall HIV funding from international donors. Approximately 70% of Namibia’s HIV funding is generated locally [42]. Because donors’ financial commitments may not be stable [43], the Ghana government should explore innovative financing mechanisms to strengthen and expand domestic funding through the Ghana AIDS Trust while strengthening partnerships with international donors to help ensure consistent funding [8].
8. Data Quality and Monitoring: robust monitoring and evaluation systems are essential to track progress towards the targets and identify areas that require intervention. However, challenges in data quality, completeness, and timeliness pose barriers to effective monitoring and decision-making [50]. Strengthening data systems, improving data collection processes, and enhancing the capacity of healthcare workers to collect and analyse data are crucial for evidence-based interventions. Routine monitoring of population-level progress towards the UNAIDS 2025 target of 95-95-95 (testing, treatment, and viral suppression) can help drive public health programmes and increase programmatic efficiencies [63]. Data collection, monitoring, and analysis should also be directed towards specific HIV programme interventions and activities, including supportive supervisory visits to service delivery sites, mentoring initiatives such as ‘Community Adolescent Treatment Supporters’ and ‘Mentor Mothers’, and performance evaluations. This approach ensures accountability, transparency, and the overall success of programmes.
9. Multi-Sectoral Collaboration: the Ghana AIDS Commission should spearhead collaboration among various stakeholders, including government agencies, civil society organisations, healthcare providers, affected communities, and international partners, as this is crucial. Strong coordination, information sharing, and joint efforts are necessary to implement evidence-based interventions, maximise resources, and address the complex challenges associated with HIV/AIDS [25].

## Conclusion

Achieving the UNAIDS treatment target for HIV/AIDS by 2025 in Ghana is an ambitious but attainable reality. While Ghana has made significant progress in its HIV/AIDS response, challenges and barriers persist. However, the country has demonstrated a strong commitment to addressing these challenges through a comprehensive and multi-faceted approach. While challenges exist, Ghana has the potential to achieve the UNAIDS treatment target for HIV/AIDS by 2025. The country’s progress in HIV testing, ART coverage, viral suppression rates, and strategic interventions demonstrates its commitment to ending the AIDS epidemic. With continued dedication, collaboration, and a comprehensive approach, Ghana can overcome barriers, strengthen its HIV/AIDS response, and ultimately achieve the UNAIDS treatment target, improving the health and well-being of individuals living with HIV/AIDS and contributing to a healthier future for all Ghanaians.

Overall, achieving the UNAIDS treatment target in Ghana is not a myth but a tangible reality within reach, requiring collective effort and sustained commitment from all stakeholders involved in the HIV/AIDS response.

## Paper context

The United Nations has set ambitious treatment targets to combat the global HIV/AIDS epidemic by 2030. Ghana has made significant progress towards the treatment targets by 2025 but faces substantial challenges. Through a combination of prevention, testing, treatment, and care services, the country aims to reduce new infections, ensure early diagnosis, and improve the quality of life for people living with HIV/AIDS. With sustained efforts, Ghana can make significant strides in ending the AIDS epidemic.
